# Quality of Life after Mastectomy with or without Breast Reconstruction and Breast-Conserving Surgery in Breast Cancer Survivors: A Cross-Sectional Study at a Tertiary Hospital in Ghana

**DOI:** 10.3390/curroncol31060224

**Published:** 2024-05-24

**Authors:** Josephine Nsaful, Edmund Tetteh Nartey, Florence Dedey, Antoinette Bediako-Bowan, Rita Appiah-Danquah, Kwame Darko, Levi Nii Ayi Ankrah, Cynthia Akli-Nartey, Jessie Yaoteokor Annan, Jessica Dei-Asamoa, George Amanquanor Ahene-Amanquanor, Joe-Nat Clegg-Lamptey

**Affiliations:** 1 Department of Surgery, University of Ghana Medical School, College of Health Sciences, University of Ghana, Accra GA-221-1570, Ghana; fdedey@ug.edu.gh (F.D.); abediako-bowan@ug.edu.gh (A.B.-B.); clegglamptey@ug.edu.gh (J.-N.C.-L.); 2 Department of Surgery, Korle Bu Teaching Hospital, Accra GA-221-1570, Ghana; ritaappiahdanquah@yahoo.com (R.A.-D.); opokua20@gmail.com (C.A.-N.); jessannan@yahoo.com (J.Y.A.); jesakwe@gmail.com (J.D.-A.); george.ahene@gmail.com (G.A.A.-A.); 3 Centre for Tropical Clinical Pharmacology & Therapeutics, University of Ghana Medical School, Accra GA-221-1570, Ghana; etnartey@ug.edu.gh; 4 National Reconstructive Plastic Surgery and Burns Centre, Korle Bu Teaching Hospital, Accra GA-221-1570, Ghana; kwame.darko15@gmail.com (K.D.); niidee@yahoo.com (L.N.A.A.)

**Keywords:** breast cancer, quality of life, EORTC QLQ, mastectomy, breast reconstruction, breast conservation

## Abstract

(1) Background: Breast cancer is the leading malignancy worldwide, and in Ghana, it has a poor overall survival rate. However, approximately 50% of cases are cases of early-stage disease, and with advances in breast cancer treatment and improvements in survival, quality of life (QOL) is becoming as important as the treatment of the disease. (2) Methodology: This was a cross-sectional study of survivors who had breast-conserving surgery (BCS), mastectomy only (M) and mastectomy with breast reconstruction (BRS) from 2016 to 2020 at a tertiary hospital in Ghana, comparatively assessing their QOL using *EORTC QLQ C-30* and *EORTC QLQ BR-23.* (3) Results: The study participants had an overall *global health status* (GHS) median score of 83.3 [IQR: 66.7–91.7] with no significant differences between the surgery types. The BRS group had statistically significant lower median scores for the *functional scale* (82.8 and 51.0) and the highest scores for the *symptomatic scale* (15.7 and 16.5). *Body image* was significantly lowest for the BRS group (83.3) [68.8–91.7] and highest (100) [91.7–100] for the BCS group (*p* < 0.001). (4) Conclusion: There is a need to develop support systems tailored at improving the QOL of breast cancer survivors taking into consideration the type of surgery performed.

## 1. Introduction

Breast cancer (BC) is the leading malignancy worldwide. In Ghana, it is the leading malignancy as well as the leading cause of cancer mortality [1]. BC has a 5-year survival rate exceeding 90% in high-income countries (HICs) and 40–66% in low-to-middle-income countries (LMICs) [2].

Ghana has a reported overall 5-year survival of 47.65%, similar to most LMICs. However, those with early-stage disease (Stage 0, I) have a reported 5-year survival of 91.94% [3]. The overall poor survival is attributable to the large proportion of those with advanced disease, as stage 4 disease in Ghana has survival rates of only 15.09% [3].

With improvement in early reporting, advances in BC treatment and subsequent improvements in survival, quality of life (QOL) has become an important consideration in management. Treatment of the disease takes a toll on the patient physically, emotionally, socially and, in LMICs, financially. According to the WHO, quality of life is “an individual’s perception of their position in life in the context of the culture and value systems in which they live and in relation to their goals, expectations, standards and concerns” [4].

The objective assessment of treatment outcomes is important in assessing the impact of the risks versus benefits to the patient. There are various QOL assessment tools available. The European Organisation for Research and Treatment of Cancer core questionnaire, EORTC QLQ-C30, is one of the well-known and widely used assessment tools; the EORTC QLQ-BR23 is its BC-specific supplementary module. The EORTC QLQ-C30 assesses global, functional and symptom domains of cancer patients; the EORTC QLQ-BR23 assesses BC-specific functional and symptom domains [5].

BC treatments can affect a patient’s quality of life. Some may have short-term effects on QOL, while others may be more long-lasting. Surgery in particular can be radical, affecting a woman’s body image and self-confidence. Long-term side effects such as lymphoedema, shoulder stiffness and chronic pain may also follow surgery [6]. With large numbers of cases of advanced-stage disease in Ghana and other LMICs, more mastectomies are performed compared to breast-conserving surgeries. Stage for stage, the choice of surgery does not affect survival outcomes; this rather depends on the various adjuvant therapies [7]. In recent years, breast reconstruction has become available as an adjunct procedure to mastectomy, though the cost and acceptance of this procedure makes it less commonly performed in LMICs [8].

The concept of QOL has not been adequately explored in our cultural context. This paper compares outcomes in QOL in BC survivors who had mastectomy only (M), mastectomy with breast reconstruction (BRS) and breast-conserving surgery (BCS) using the EORTC QLQ-C30 and EORTC QLQ-BR23 QOL tools. To the best of our knowledge, this is the first study in Ghana to investigate the various aspects of QOL relating to the type of BC surgery, including BRS.

## 2. Materials and Methods

This was an unmatched cross-sectional study of BC survivors who had surgery from 2016 to 2020, 2 years or more prior to the study conducted in 2022, at the Korle Bu Teaching Hospital (KBTH). The KBTH is a tertiary hospital and one of three centres in Ghana that offer comprehensive breast cancer care (including diagnostic [radiology, pathology including immunohistochemistry], surgery [including breast reconstruction], clinical and radiation oncology services) in the country.

A database of patients who had surgery over this period was created from the admission record books of the breast surgical ward. Surgeries included were breast-conserving surgery and mastectomy with or without immediate breast reconstruction. Delayed breast reconstruction, male patients and patients on active breast cancer treatment (chemotherapy and radiotherapy) were excluded. Those on hormonal therapy were, however, included. Patients or their next of kin were contacted by telephone and interviews scheduled. Participants were invited to the hospital to be interviewed in person. Those who could not come to the hospital were given the option of a telephone interview. Written informed consent was taken for in-person interviews and a telephone consent script was read out over the phone. Interviews were conducted mostly in English, with a few conducted in the dominant Twi local language by the same research assistant to limit variability. A quantitative research approach was utilized to obtain information on the QOL of BC survivors.

The first part of the study questionnaire covered data on participant demographics and clinical stage at diagnosis, which was obtained from patients’ records. Participants were also asked about satisfaction with the appearance of their breast/chest wall. The second part was the, EORTC QLQ C-30 and EORTC QLQ BR-23. The EORTC QLQ C-30, composed of both multi-item scales and single-item measures, includes five functional scales on physical, role, emotional, social and cognitive functioning; three symptom scales measuring fatigue, pain and emesis; a global health status (overall QOL) scale; and six single items assessing financial impact and symptoms such as dyspnoea, nausea, vomiting, appetite, diarrhoea and constipation.

The EORTC QLQ BR-23 covers the functional and symptom scales specific to BC. The functional scale consists of body image, sexual functioning, sexual enjoyment and future perspective. The symptom scale consists of side effects of systemic therapy, breast symptoms, arm symptoms and upset by hair loss.

The parameters were scored 1 to 4, and the scoring manuals of EORTC QLQ C-30 and EORTC QLQ BR-23 were applied to obtain the interpretation. High scores for the functional scales imply a high or healthy level of functioning, and high scores for the symptom scale imply a high level of symptomatology or problems.

### Statistical Analysis

Stata^®^ 14 software was used to analyse the data. Categorical variables were reported as percentages and proportions and were compared using Pearson chi-square statistics. All the scales and single-item measures range in score from 0 to 100. A high score for a functional scale represents a high/healthy level of functioning, and a high score for the global health status/QOL represents a high QOL, but a high score for a symptom scale/item represents a high level of symptomatology/problems. The raw scores were transformed to scores ranging from 0 to 100 by using the following formula [5,9]:

$$Raw\;score=RS=\left(I1+I2\ldots +In\right)/n$$

The linear transformation was applied to 0–100 to obtain the S score:

$$Functional\;scale:S=\left(\;1-\frac{RS-1}{range}\right)\times 100$$

$$Symptom\;scale:S=\left(\frac{RS-1}{range}\right)\times 100$$

The calculated median score of the transformed RS was used to compare QOL domains among the surgery groups. Sub-group analysis was conducted among the surgery groups to determine any relation between type of surgery and QoL. Statistical significance was considered at *p*-values less than 0.05.

## 3. Results

Nine hundred and ninety-four (994) patients had surgery from 2016 to 2020. A total of 494 were not reachable by phone: there was no record of telephone numbers for 95 patients, and the rest had deactivated phones or did not respond after six attempts. Of the 500 patients or their next of kin that were contacted, 202 patients were reported dead by their next of kin. Of the remaining 298, 45 did not participate for various reasons including refusal to consent, being too ill to talk, travelling out of town or a language barrier. A total of 253 survivors therefore consented to participate in the study, giving a response rate of 85% of survivors reached. Of the number interviewed, 164 (64.8%) were interviewed in-person and 89 (35.2%) over the telephone.

### 3.1. Sociodemographic and Clinical Characteristics

The overall mean age of study participants was 55.2 ± 12.7 years with an overall median [IQR] time since surgery of 48.0 [32.0–65.5] months. Of the 253 study participants, 162 (64.0%) had mastectomy without breast reconstruction (M), 24 (9.5%) had mastectomy with breast reconstruction (BRS) and 67 (26.5%) had breast-conserving surgery (BCS).

Table 1 shows the socio-demographic and clinical characteristics of participants. The mean ages of the M, BRS and BCS study participants were 52.9 (±11.8), 40.9 (±11.3) and 58.6 (±12.1) years, respectively; this was statistically significant (*p* < 0.001). Educational level was also significantly different between the three surgery groups, with 87.5% (*n* = 21) of the BRS having tertiary education (*p* < 001) (Table 1). A total of 50% (*n* = 12) of the BRS group were pre-menopausal, statistically significant from that of the M and BCS groups (*p* = 0.022) (Table 1). Employment status at diagnosis and monthly income were significantly associated with the type of surgery (*p* < 0.001). The clinical stage of BC was statistically associated with the type of surgery performed (*p* < 0.001), with 25.4% (*n* = 17) of the BCS group being stage I (Table 1).

### 3.2. Global Health Status

Table 2 shows the median [IQR] global health status (overall QOL) of the study participants. Stratified over the socio-demographic characteristics, the median *GHS* was over 50.0 in all strata and was not significantly different within each socio-demographic characteristic (*p* > 0.05). The analysis of Table 2 indicates that the median [IQR] *GHS* of the study participants with current breast metastasis (66.7 [IQR: 37.5–81.3]) was significantly lower compared with study participants without metastasis (*GHS* of 88.3) (*p* = 0.007). Similarly, participants being satisfied with how their breasts/chest looks was significantly associated with a higher *GHS* score (*p* = 0.009).

### 3.3. EORTC QLQ

Table 3 shows the *GHS* and the various QOL scales (both *QLQ-C30 and QLQ-BR23*) of the study participants stratified over the surgery groups. The study participants had an overall *GHS* median score of 83.3 [IQR: 66.7–91.7], with no statistically significant differences between the surgery types (*p* = 0.615).

#### 3.3.1. EORTC QLQ C-30

On the *QLQ-C30 scales*, the overall *functional Scale* median score was 91.7 [IQR: 82.5–96.7], and the BRS group had a statistically significant lower score (82.8 [IQR:77.3–91.7]) (*p* = 0.006) compared with the other surgery groups (Table 3). At the sub-scale level, the *emotional functioning* median score of 87.5 [IQR: 68.8–91.7] in the BRS group was significantly the lowest among the surgery types (*p* = 0.016) (Table 3)

On the *QLQ-C30 symptomatic scale*, the median score of the study participants was 8.6 [IQR: 1.9–17.3], with the BRS group having the significantly highest score of 15.7 [IQR: 4.2–25.8] compared with the other surgery groups (*p* = 0.033). On the sub-scale level, the BRS group had significant highest score among the surgery groups for *pain* (*p* = 0.029) and *insomnia* (*p* = 0.024) (Table 3).

#### 3.3.2. EORTC QLQ BR-23

On the *QLQ-BR23 functional scale*, the overall median score for the study participants was 70.8 [IQR: 54.1–83.3], with the BRS group having the statistically significant lowest median score of 51.0 [IQR: 40.1–56.8] compared with the other surgery groups of 75.0 [IQR: 58.3–85.4] (*p* = 0.003) (Table 3). On the sub-scale level, median scores for *body image* and *future perspective* were similarly lowest in the BRS group, and the BCS group had the highest *body image* of 100 [91.7–100] compared with the other surgery groups (*p* < 0.001) (Table 3). Sub-group analysis indicated that the median score for *body image* of the BCS was significantly higher (*p* = 0.004) compared with the M group, with 74.6% having the excellent score of 100 for BCS, whilst 57.4% had the excellent score of 100 for M. Of the 83 study participants with a *sexual functioning* score >0, the median *sexual enjoyment* score was 66.7 [IQR: 33.3–100] (Table 3).

On the *QLQ-BR23 symptomatic scale*, the median score was 6.9 [IQR: 2.8–13.5], with the BRS group having the statistically significant highest median score of 16.5 [IQR: 7.0–23.2] compared with the other surgery groups (*p* < 0.001). Median scores for *systemic therapy side effects*, *arm symptoms* and *breast symptoms* were all statistically significantly the highest among BRS compared with the other surgery types (*p* < 0.05) (Table 3).

## 4. Discussion

This study provides insight into the QOL of BC patients treated in Ghana who had BCS, M and BRS. The study quantitatively assessed the QOL of BC survivors according to the type of breast surgery they had. The findings reveal that there was no difference in the *GHS* of the various surgery groups. Those who had BRS, however, did have a significant difference in the domains of functionality and symptomatology. Body image was found to be better with the BCS group.

The overall mean age of study participants was 55.2 ± 12.7 years, which falls in line with other publications from Ghana [3,10]. The significant differences in cancer stage between groups reflects the role of staging in the choice of surgery: stages I and II disease are more likely to receive BCS, and stage III disease, mastectomy. The mean age for the surgery groups differed significantly, with the BRS group being younger [40.9 years (± 11.3)] and the M and BCS groups a decade older [52.9 (± 11.8) and 58.6 (± 12.1) years, respectively]. Similar age differences in surgery groups have been reported in Italy, Korea, the Netherlands and the USA [11,12,13,14]. A goal of BRS is to improve one’s self-image and self-confidence; therefore, younger women who may be more concerned about their body image are more likely to opt for BRS. Similarly, this study, like other studies, found higher education to be associated with the BRS group [11,12], implying that this group may have a better awareness and appreciation of BRS. Participants in the BRS group were all employed and in a higher income bracket than those in the M or BCS group. BRS has been associated with higher-income groups [12,13], whether covered by health insurance [12] or not [13]. In Ghana, the cost of BRS is currently not covered by the National Health Insurance Scheme and, therefore, cost may be a consideration for lower income earners in their choice of surgery. Complaints about financial difficulties were generally minimal, though notably less in the BRS group, but not significantly associated with a particular surgery group (Table 3).

The *global health status (GHS)* scores which reflect the overall QOL of the participants were generally high (83.3 [IQR: 66.7–91.7]) for all surgery groups. Some studies utilizing the *EORTC QLQ C-30* and *QLQ BR-23* report lower *GHS* scores [13,15,16,17,18], whilst the high scores found in this study are very similar to those reported from the Netherlands [14]. In a community where the general outlook towards BC is rather grim and expected to culminate in death, participants will have higher *GHS* scores because they feel and function better than anticipated. [19]. The *GHS* scores were not found to be associated with any of the sociodemographic factors, clinical stage, side of surgery or time since surgery, but they were significantly lower in those who had disease progression after surgery to metastatic disease and higher in those who were satisfied with the appearance of their breasts/chest (Table 3). In other studies, however, *GHS* has been found to be higher in younger women [15,16], those with a higher income and those for which a longer time has passed since surgery [16,20]. It is interesting that a study found higher *GHS* scores among BC patients than the general public [21]. A meta-analysis of studies utilizing the EORTC tools found the lowest QOL scores from South America, followed by Africa [22]. This suggests that QOL should be interpreted within relevant cultural contexts.

There was no difference in *GHS* between surgery groups, as reported by several others [11,13,14,16,17], which means a good quality of life can be achieved irrespective of the type of surgery one has. A QOL study at the same institution also found no difference in QOL when patients were categorized according to treatment modalities (not limited to surgery) [23]. Some studies, however, do report a better QOL for BCS relative to M [24,25], and others report better QOL for M than BCS [26]. Although a study by Janni et al. objectively also found no difference in *GHS,* post-mastectomy patients were more likely to be dissatisfied with cosmetic outcomes and were more emotionally disturbed than BCS patients [27].

This study reports a lower *functional scale* score for the BRS group due to significantly lower *body image* (though the median score of 83.3 [68.8–91.7] is reasonably high) and *future perspective* scores in the sub-scale analysis. The median *body image* scores for BCS and M were high, implying both groups were very satisfied with their self-image, but the *body image* score of BCS was significantly higher compared with that of M. A meta-analysis by Elvin et al. (2019) found higher *body image* and *future perspective* scores associated with BCS as compared to mastectomy [24], though that study did not include BRS. There is a widely held misconception in the Ghanaian community that mastectomy leads to death and that BC is not survivable [19]. Perhaps women who had M in the Ghanaian cultural context, where BC mortality is high among patients reporting with locally advanced disease, may not have been pre-occupied with body image, having survived the disease and surgery.

The median *future perspective* score of 33.3 was extremely low for the BRS group. The question asked was, “During the past week, were you worried about your health in the future?” These concerns are most likely due to non-cancer-related issues after BRS. Fear of implant-related complications, repeat surgical procedures and the implant causing a new cancer are issues dealt with by BRS patients [28,29]. These issues need to be explored qualitatively in the Ghanaian cultural context to understand and address the reasons for such low scores. This study found excellent *future perspective* scores for both M and BCS groups, particularly with the M group. This has also been found to be higher for M patients in India [25]. Once the breast has been removed, there is less fear of local recurrence.

The BRS group also had the lowest *functional scale* score on the *EORTC QLQ C-30* due to having the lowest scores on *emotional functioning*. The *symptomatic scale* scores were significantly highest, also implying a poorer QOL for the BRS group compared with the other surgery groups due to higher *pain*, *insomnia*, *systemic therapy side effects*, *arm symptoms*, and *breast symptoms* scores. Notably the BRS group’s sexual *enjoyment* scores were the lowest but not statistically significant. *Sexual function* and *sexual enjoyment* have been found to be significantly associated with age but not with type of surgery [26].

*Systemic therapy side effects* for the BCS group were higher than for the M group, possibly from the adjuvant radiation therapy received by this group. A meta-analysis, however, found lower *systemic therapy side effects* scores associated with BCS [24]. QOL is a function of time; emotional disturbance and anxiety reduces over time and general well-being increases [29]. Various EORTC sub-scales have been found to change over time [30].

The essence of BRS is to improve QOL. In our setting, like in other LMICs, breast reconstruction is a relatively new procedure with low acceptance and is not routinely offered to all patients [8]. It is sometimes considered only when the patient has difficulty accepting mastectomy. Such patients are usually perceived to be “emotional”. Perhaps pre-existing emotional challenges with accepting diagnosis and surgery may contribute to the lower *emotional functioning* and possibly higher *pain* and *insomnia* scores. Indeed, it has been reported that women who have BRS have a lower QOL and more mood disturbances, corroborating the findings of this study [31]. However, a limitation of this study is that QOL was not assessed before surgery as a baseline to determine if the differences were due to treatment/surgery or were pre-existing. These findings are of clinical importance, and the concerns of the BRS group should not be taken lightly. This group of patients is composed of young, educated, gainfully employed women who need support to overcome challenges not only of a BC diagnosis but in coping with body image, emotions, lingering treatment side effects and future health concerns. Efforts tailored to address these concerns will improve their QOL.

This study has attempted to fill the literature gap on QOL outcomes of the various surgical options in LMICs. Based on the findings of BC survivors treated in Ghana, clinicians, in discussing surgical options with patients, should indicate that a good QOL is achievable irrespective of the choice of breast surgery. Once oncological principles are satisfied, decision making should be individualized. This is particularly important in our setting where patients refuse treatment, believing that surgery, particularly mastectomy, will maim and deform them, leaving them functionless and as social outcasts. These findings will serve as evidence-based decision-making aids for patients and clinicians. There is a need to develop a comprehensive approach in counselling patients before and after surgery; this should include psychological evaluation and support pre-operatively and in the long term to cater for the differences in certain aspects of QOL, taking into consideration individual sociodemographic and clinical peculiarities. Additionally, for those who opt for breast reconstruction, clinicians should identify the peculiar issues underlying the challenges associated with body image and emotional function. Future perspective and support programs should teach coping mechanisms to address these challenges. There is therefore the need for qualitative research to understand these outcomes within our cultural contexts and quantitatively use a combination of various assessment tools to comprehensively cover various aspects of QOL. This will shape the development of interventions designed to support BC patients, culminating in an improvement in QOL after treatment.

The limitations of this study include possible variation introduced from interviews carried out in the local language. The small sample size, due to the high mortality of the disease in LMICs and challenges in following up patients due to incomplete records, also limits the generalizability of the study findings. The Post hoc power analysis indicates that the size of the M group (N = 162) and BCS group (N = 67) provided the study with a power of 17.0% to detect a difference in QOL between the groups of at least 15 points, and therefore, the two groups had similar QOL (*GHS*) scores within a margin of 15 points. The numbers in the BRS group were particularly small; however, this is a reflection of the low patronage of this surgical option. A multicentre study would improve the sample size and generalizability of the results. In spite of these limitations, we are of the opinion that this study will be of great benefit to clinicians in our setting and the sub-region.

## 5. Conclusions

In summary, this study found no difference in overall QOL between M, BRS and BCS as body image scores were all high. The BRS group, however, had significantly lower functioning due to relatively lower self-body image and mostly concerns about their future health. This group also had low emotional function and more post-treatment symptomatology due to pain and insomnia. Self-body image for both M and BCS was high, but that for the BCS group was the highest.

There is a need to develop support systems tailored at improving the QOL of breast cancer patients to guide the choice of surgery and help them cope psychologically, emotionally and physically in the long term.

## Figures and Tables

**Table 1:** Socio-demographic and clinical characteristics of study participants.

Characteristic		Mastectomy (M)	Mastectomy (with BRS)	Breast-Conserving Surgery (BCS)	*p*-Value
		*n*, % ^1^	*n*, % ^1^	*n*, % ^1^	
Age, mean (±SD)		52.9 ± 11.8	40.9 ± 11.3	58.6 ± 12.1	**<0.001**
Marital status		*N = 162*	*N = 24*	*N = 67*	0.111
	Married	76, (46.9)	11 (45.8)	38 (56.7)	
	Single	25, (15.4)	8 (33.3)	10 (14.9)	
Divorced/Separated		24 (14.8)	4 (16.7)	6 (9.0)	
	Widowed	37 (22.8)	1 (4.2)	13 (19.4)	
Educational level		*N = 162*	*N = 24*	*N = 67*	**<0.001**
	Tertiary	63 (38.9)	21 (87.5)	20 (29.9)	
	Secondary	54 (33.3)	2 (8.3)	32 (47.8)	
	Primary	29 (17.9)	1 (4.2)	13 (19.4)	
	None	16 (9.9)	0 (0)	2 (3.0)	
Religion		*N = 162*	*N = 24*	*N = 67*	0.806
	Christian	150 (92.6)	22 (91.7)	63 (94.0)	
	Muslim	12 (7.4)	2 (8.3)	4 (6.0)	
Menopausal stage		*N = 162*	*N = 24*	*N = 67*	**0.022**
Pre-menopausal		39 (24.1)	12 (50.0)	14 (20.9)	
Post-menopausal		123 (75.9)	12 (50.0)	53 (79.1)	
Employment status at diagnosis		*N = 162*	*N = 24*	*N = 67*	**<0.001**
	Employed	124 (76.5)	24 (100)	41 (61.2)	
Unemployed		38 (23.5)	0 (0.0)	26 (38.8)	
Monthly income		*N=162*	*N = 24*	*N = 67*	**<0.001**
≤GHS 500		53 (32.7)	2 (8.3)	21 (31.3)	
GHS 501–1000		49 (30.3)	0 (0.0)	20 (29.9)	
GHS 1001–2000		22 (13.6)	7 (29.2)	15 (22.4)	
GHS 2001–5000		31 (19.1)	13 (54.2)	10 (14.9)	
GHS >5000		7 (4.3)	2 (8.3)	1 (1.5)	
Clinical stage of breast cancer at diagnosis		*N=162*	*N = 24*	*N = 67*	**<0.001**
Stage I		8 (4.9)	1 (4.2)	17 (25.4)	
Stage II		51 (31.5)	5 (20.8)	32 (47.8)	
Stage III		56 (34.6)	13 (54.2)	9 (13.4)	
Unknown		47 (29.0)	5 (20.8)	9 (13.4)	
Duration since surgery		*N=162*	*N = 24*	*N = 67*	
25–36 months		59 (36.4)	4 (16.7)	14 (20.9)	
37–48 months		33 (16.7)	6 (25.0)	12 (17.9)	
49–60 months		27 (16.7)	10 (41.7)	12 (17.9)	
≥61 months		43 (26.5)	4 (16.7)	29 (43.3)	

^1^ Column percentages; BRS = breast reconstruction surgery; USD 1 = GHS 10.0.

**Table 2:** Clinical parameters associated with global health status.

Characteristic		Global Health Status, Median [IQR]	*p*-Value
Age			0.552
<50 years		83.3 [66.7–89.6]	
≥50 years		83.3 [66.7–91.7]	
Clinical stage of breast cancer			0.061
Stage I		83.3 [75.0–91.7]	
Stage II		75.0 [66.7–91.7]	
Stage III		75.0 [66.7–91.7]	
Unknown		83.3 [75.0–91.7]	
Surgery same side as dominant hand			0.087
Yes		83.3 [66.7–85.4]	
No		83.3 [66.7–91.7]	
Current metastatic status			**0.002**
	Non-metastatic	83.3 [66.7–91.7]	
	Metastatic	66.7 [37.5–81.3]	
Satisfaction with how breasts/chest looks			**0.009**
	Yes	83.3 [66.7–91.7]	
	No	70.8 [58.3–83.3]	
Time since surgery			0.867
	24–36 months	83.3 [66.7–91.7]	
	37–48 months	83.3 [66.7–91.7]	
	49–60 months	83.3 [66.7–83.3]	
	≥61 months	83.3 [66.7–91.7]	

BRS = breast reconstruction surgery; IQR = interquartile range.

**Table 3:** Type of breast surgery in study participants associated with global health status and QOL domains.

Characteristic		All Surgery Types	Mastectomy (M)	Mastectomy (with BRS)	Breast Conservation Surgery (BCS)	*p*-Value *
QLQ-C30 global health status, median [IQR]		83.3 [66.7–91.7]	83.3 [66.7–91.7]	79.2 [66.7–83.3]	83.3 [66.7–91.7]	0.615
QLQ-C30 functional scale, median [IQR]		91.7 [82.5–96.7]	92.7 [83.6–97–3]	82.8 [77.3–91.7]	92.3 [83.7–96.7]	**0.006**
	Physical functioning	93.3 [86.7–100]	93.3 [80.0–100]	86.7 [86.7–100]	93.3 [86.7–100]	0.423
	Role functioning	100 [83.3–100]	100 [100]	91.7 [66.7–100]	100 [83.3–100]	0.107
	Emotional functioning	91.7 [75.0–100]	100 [83.3–100]	87.5 [68.8–91.7]	91.7 [75.0–100]	**0.016**
	Cognitive functioning	100 [83.3–100]	100 [79.2–100]	83.3 [70.8–100]	83.3 [83.3–100]	0.302
	Social functioning	100 [100]	100 [100]	100 [66.7–100]	100 [100]	0.091
QLQ-C30 symptom scale, median [IQR]		8.6 [1.9–17.3]	5.2 [1.2–15.6]	15.7 [4.2–25.8]	11.1 [1.9–16.7]	**0.033**
	Fatigue	11.1 [0.0–22.2]	0.0 [0.0–22.2]	22.2 [0.0–41.7]	11.1 [0.0–22.2]	0.061
	Nausea and vomiting	0.0 [0.0–0.0]	0.0 [0.0–0.0]	0.0 [0.0–0.0]	0.0 [0.0–0.0]	0.444
	Pain	0.0 [0.0–33.3]	0.0 [0.0–33.3]	25.0 [4.2–45.8]	0.0 [0.0–33.3]	**0.029**
	Dyspnoea	0.0 [0.0–0.0]	0.0 [0.0–0.0]	0.0 [0.0–33.3]	0.0 [0.0–0.0]	0.382
	Insomnia	0.0 [0.0–33.3]	0.0 [0.0–0.0]	33.3 [0.0–33.3]	0.0 [0.0–33.3]	**0.024**
	Appetite loss	0.0 [0.0–0.0]	0.0 [0.0–0.0]	0.0 [0.0–0.0]	0.0 [0.0–0.0]	0.539
	Constipation	0.0 [0.0–0.0]	0.0 [0.0–0.0]	0.0 [0.0–0.0]	0.0 [0.0–0.0]	0.519
	Diarrhoea	0.0 [0.0–0.0]	0.0 [0.0–0.0]	0.0 [0.0–0.0]	0.0 [0.0–0.0]	0.321
	Financial difficulties	0.0 [0.0–66.7]	0.0 [0.0–66.7]	0.0 [0.0–33.3]	0.0 [0.0–66.7]	0.836
QLQ-BR23 functional scale, median [IQR]		70.8 [54.1–83.3]	75.0 [58.3–85.4]	51.0 [40.1–56.8]	75.0 [54.7–83.3]	**0.003**
Body image		100 [83.3–100]	100 [75.0–100]	83.3 [68.8–91.7]	100 [91.7–100]	**<0.001**
Future perspective		100 [33.3–100]	100 [66.7–100]	33.3 [0.0–66.7]	100 [33.3–100]	**<0.001**
Sexual functioning		0.0 [0.0–33.3]	0.0 [0.0–33.3]	33.3 [0.0–50.0]	0.0 [0.0–16.7]	0.109
Sexual enjoyment (N = 83)		66.7 [33.3–100]	66.7 [33.3–100]	50.0 [25.0–66.7]	66.7 [16.7–100]	0.164
QLQ-BR23 symptom scale, median [IQR]		6.9 [2.8–13.5]	6.3 [2.4–11.9]	16.5 [7.0–23.2]	7.1 [2.8–14.1]	**<0.001**
Systemic therapy side effects		4.8 [0.0–14.3]	4.8 [0.0–14.3]	11.9 [4.8–27.4]	9.5 [4.8–19.0]	**0.004**
	Upset by hair loss	0.0 [0.0–0.0]	0.0 [0.0–0.0]	0.0 [0.0–0.0]	0.0 [0.0–0.0]	0.937
	Arm symptoms	11.1 [0.0–22.2]	11.1 [0.0–22.2]	22.2 [11.1–44.4]	11.1 [0.0–22.2]	**<0.001**
	Breast symptoms	8.3 [0.0–16.7]	8.3 [0.0–16.7]	16.7 [8.3–22.9]	0.0 [0.0–16.7]	**0.012**

* Kruskal–Wallis equality-of-populations rank test between the surgery groups (M, BRS and BCS); BRS = breast reconstruction surgery; IQR = interquartile range.

## Data Availability

The data presented in this study is available at https://doi.org/10.6084/m9.figshare.25425760 (accessed on 17 March 2024).

## References

[1] IARC Global Cancer Observatory—Ghana. https://gco.iarc.who.int/media/globocan/factsheets/populations/288-ghana-fact-sheet.pdf.

[2] Breast Cancer Inequities. https://www.who.int/initiatives/global-breast-cancer-initiative/breast-cancer-inequities.

[3] Mensah A.C., Yarney J., Nokoe S.K., Opoku S., Clegg-Lamptey J.N. (2016). Survival Outcomes of Breast Cancer in Ghana: An Analysis of Clinicopathological Features. Open Access Library.

[4] WHOQOL—Measuring Quality of Life|The World Health Organization. https://www.who.int/tools/whoqol.

[5] Aaronson N., Ahmedzai S., Bergman B., Bullinger M., Cull A., Duez N.J., Filiberti A., Flechtner H., Fleishman S., de Haes J.C. (1993). The European Organisation for Research and Treatment of Cancer QLQ-C30: A quality-of-life instrument for use in international clinical trials in oncology. J. Natl. Cancer Inst..

[6] Lovelace D.L., McDaniel L.R., Golden D. (2019). Long-Term Effects of Breast Cancer Surgery, Treatment, and Survivor Care. J. Midwifery Womens Health.

[7] Onitilo A.A., Engel J.M., Stankowski R.V., Doi S.A.R. (2015). Survival Comparisons for Breast Conserving Surgery and Mastectomy Revisited: Community Experience and the Role of Radiation Therapy. Clin. Med. Res..

[8] Nair N.S., Penumadu P., Yadav P., Sethi N., Kohli P.S., Shankhdhar V., Jaiswal D., Parmar V., Hawaldar R.W., Badwe R.A. (2021). Awareness and Acceptability of Breast Reconstruction Among Women with Breast Cancer: A Prospective Survey. JCO Glob. Oncol..

[9] csi (2017). EORTC—Quality of Life. Manuals. https://qol.eortc.org/manuals/.

[10] Ssentongo P., Oh J.S., Amponsah-Manu F., Wong W., Candela X., Acharya Y., Ssentongo A.E., Dodge D.G. (2022). Breast Cancer Survival in Eastern Region of Ghana. Front. Public Health.

[11] Spatuzzi R., Vespa A., Lorenzi P., Miccinesi G., Ricciuti M., Cifarelli W., Susie M., Fabriziof T., Ferrarig M.G., Ottaviani M. (2016). Evaluation of Social Support, Quality of Life, and Body Image in Women with Breast Cancer. Breast Care.

[12] Jagsi R., Li Y., Morrow M., Janz N., Alderman A., Graff J., Hamilton A., Katz S., Hawley S. (2015). Patient-reported Quality of Life and Satisfaction with Cosmetic Outcomes after Breast Conservation and Mastectomy with and without Reconstruction: Results of a Survey of Breast Cancer Survivors. Ann. Surg..

[13] Sun Y., Kim S.W., Heo C.Y., Kim D., Hwang Y., Yom C.K., Kang E. (2014). Comparison of Quality of Life Based on Surgical Technique in Patients with Breast Cancer. Jpn. J. Clin. Oncol..

[14] Lagendijk M., van Egdom L.S.E., van Veen F.E.E., Vos E.L., Mureau M.A.M., van Leeuwen N., Hazelzet J.Y.A., Lingsma H.F., Koppert L.B. (2018). Patient-Reported Outcome Measures May Add Value in Breast Cancer Surgery. Ann. Surg. Oncol..

[15] Imran M., Al-Wassia R., Alkhayyat S.S., Baig M., Al-Saati B.A. (2019). Assessment of quality of life (QoL) in breast cancer patients by using EORTC QLQ-C30 and BR-23 questionnaires: A tertiary care center survey in the western region of Saudi Arabia. PLoS ONE.

[16] Tsai H.Y., Kuo R.N.C., Chung K.P. (2017). Quality of life of breast cancer survivors following breast-conserving therapy versus mastectomy: A multicenter study in Taiwan. Jpn. J. Clin. Oncol..

[17] Martins T.N.d.O., Santos L.F.d., Petter G.d.N., Ethur J.N.d.S., Braz M.M., Pivetta H.M.F. (2017). Immediate breast reconstruction versus non-reconstruction after mastectomy: A study on quality of life, pain and functionality. Fisioter. Pesqui..

[18] Abebe E., Demilie K., Lemmu B., Abebe K. (2020). Female Breast Cancer Patients, Mastectomy-Related Quality of Life: Experience from Ethiopia. Int. J. Breast Cancer.

[19] Clegg-Lamptey J., Dakubo J.C.B., Attobra Y.N. (2009). Psychological aspects of breast cancer treatment in Accra, Ghana. East. Afr. Med. J..

[20] Cohen L., Hack T.F., de Moor C., Katz J., Goss P.E. (2000). The effects of type of surgery and time on psychological adjustment in women after breast cancer treatment. Ann. Surg. Oncol..

[21] Finck C., Barradas S., Zenger M., Hinz A. (2018). Quality of life in breast cancer patients: Associations with optimism and social support. Int. J. Clin. Health Psychol..

[22] Biparva A.J., Raoofi S., Rafiei S., Kan F.P., Kazerooni M., Bagheribayati F., Masoumi G.M., Doustmehraban M., Sanaei M., Zarabbal F. (2023). Quality of life in breast cancer: Systematic review and meta-analysis. BMJ Support. Palliat. Care.

[23] Kyei K., Siaw D., Opoku S., Antwi W.K., Tagoe S. (2014). Assessment on the quality of life of breast cancer patients undergoing radiation treatment in Ghana. World J. Psychosoc. Oncol..

[24] Ng E.T., Ang R.Z., Tran B.X., Ho C.S., Zhang Z., Tan W., Bai Y., Zhang M., Tam W.W., Ho R.C. (2019). Comparing Quality of Life in Breast Cancer Patients Who Underwent Mastectomy versus Breast-Conserving Surgery: A Meta-Analysis. Int. J. Environ. Res. Public Health.

[25] Cherian K., Acharya N.R., Bhargavan R.V., Augustine P., Krishnan J.K.M. (2022). Quality of Life Post Breast Cancer Surgery: Comparison of Breast Conservation Surgery versus Modified Radical Mastectomy in a Developing Country. S. Asian J. Cancer.

[26] Alvarez-Pardo S., Romero-Pérez E.M., Camberos-Castañeda N., de Paz J.Y.A., Horta-Gim M.A., González-Bernal J.J., Mielgo-Ayuso J., Simón-Vicente L., Fernández-Solana J., González-Santos J. (2022). Quality of Life in Breast Cancer Survivors in Relation to Age, Type of Surgery and Length of Time since First Treatment. Int. J. Environ. Res. Public Health.

[27] Janni W., Rjosk D., Dimpfl T.H., Haertl K., Strobl B., Hepp F., Hanke A., Bergauer F., Sommer H. (2001). Quality of life influenced by primary surgical treatment for stage I–III breast cancer-long-term follow-up of a matched-pair analysis. Ann. Surg. Oncol..

[28] Possible Problems with Breast Reconstruction. https://www.cancerresearchuk.org/about-cancer/breast-cancer/treatment/surgery/breast-reconstruction/possible-problems.

[29] Nissen M., Swenson K., Kind E. (2002). Quality of life after postmastectomy reconstruction. Oncol. Nurs. Forum..

[30] Dahlui M., Azzani M., Taib N.A., Hoong S.M., Jamaris S., Islam T. (2023). Breast conserving surgery versus mastectomy: The effect of surgery on quality of life in breast cancer survivors in Malaysia. BMC Womens Health.

[31] Nissen M.J., Swenson K.K., Ritz L.J., Farrell J.B., Sladek M.L., Lally R.M. (2001). Quality of life after breast carcinoma surgery: A comparison of three surgical procedures. Cancer.

